# The safety of surgical technique for ileostomy and colostomy in preventing parastomal hernias: an in vitro experimental simulation study

**DOI:** 10.1186/s13037-021-00302-9

**Published:** 2021-07-26

**Authors:** Peter C. Ambe

**Affiliations:** 1 Department of General Surgery, Visceral Surgery and Coloproctology, Vinzenz-Pallotti-Hospital Bensberg, Vinzenz-Pallotti-Str. 20-24, Bensberg, 51429 Germany; 2 Department of Medicine, Chair of Surgery II, Witten/Herdecke University, Witten, Germany

**Keywords:** Parastomal hernia, Cruciate incision, Circular excision, Pressure development

## Abstract

**Background:**

Parastomal hernia (PH) is a common long-term complication in persons with an ostomy. Although the cause of PH may be multifactorial, the surgical technique employed for the creation of a stoma may be a risk factor for the development of PH. The traditional technique of cruciate fascia incision may predispose to increased pressure zones at the ostomy exit site, thereby increasing the risk of PH. A circular excision of the abdominal fascia at the ostomy exit site enables a uniform pressure distribution, thereby reducing the risk of PH. This hypothesis was tested in this in vitro experimental simulation study.

**Methods:**

The effect of the surgical technique for ostomy creation on the risk of PH development was investigated in this in vitro experimental simulation study. The pressure development at the stoma site was compared for the traditional cruciate incision vs. circular fascia excision.

**Results:**

The pressure at the ostomy site was about four-times higher in the tradition cruciate incision technique compared to the circular excision technique. This finding was independent of unilateral (e.g. peritoneal) pressure application.

**Conclusion:**

The main finding from this study suggests that the traditional cruciate incision of the abdominal fascia for the creation of an intestinal ostomy predisposes to increased pressures at the ostomy site, thus increasing the risk of PH. This effect is not seen in the experimental setting following a circular excision of the fascia. Thus, this surgical aspect may be adopted as a possible means of reducing the risk of parastomal hernia in patients undergoing ostomy surgery.

## Introduction

Parastomal hernia (PH) is a well-known long-term complication following the creation of an intestinal stoma. The incidence of PH has been reported to be as high as 80%, mostly depending on ostomy type and duration [1–5]. The diagnosis of PH is mostly clinical. However, small PH in obese patients may be difficult to diagnose. Abdominal imaging including ultrasound sonography and computed tomography (Ct) may increase diagnostic yield [6, 7]. The extent of PH can be graded using a variety of classification systems [8, 9]. The European Hernia Association recommends a classification system based on Ct imaging [10]. The classification considers the present of incisional hernias and bowel involvement with regard to the hernia sac.

While small PH mostly go unnoticed, large protruding PH render ostomy dressing difficult. Besides, abdominal pain, poor stoma function and difficulties with ostomy dressing may negatively affect the quality of life (QoL) [11]. Bowel obstruction with the need for emergency surgery and bowel resection are known complications of PH [12].

Although the etiology of PH is usually multifactorial, surgical and patient-dependent risk factors can be readily identified [13, 14]. While some patient-dependent factors may not easily correctable, paying attention to surgical details may be crucial in reducing the rate of PH [15, 16]. This is especially true with regard to the technique and size of the fascial incision [17].

Traditionally, the fascia is incised in a cruciate fashion allowing the smooth passage of two fingers [18]. It can be hypothesized that this traditional technique predisposes to high pressure development at the edges of the cruciate incision thereby promoting the development of parastomal hernia. Performing a circular excision of the fascia may lead to a more uniform pressure distribution across the abdominal wall, which may be associated with a reduction in the risk of PH development. This report summarizes the findings from an in vitro experimental simulation study based on the above hypothesis. The cruciate facial incision (traditional technique) is compared with the circular fascia excision (experimental technique) with regard to pressure development.

## Methods

**Fig. 1:**
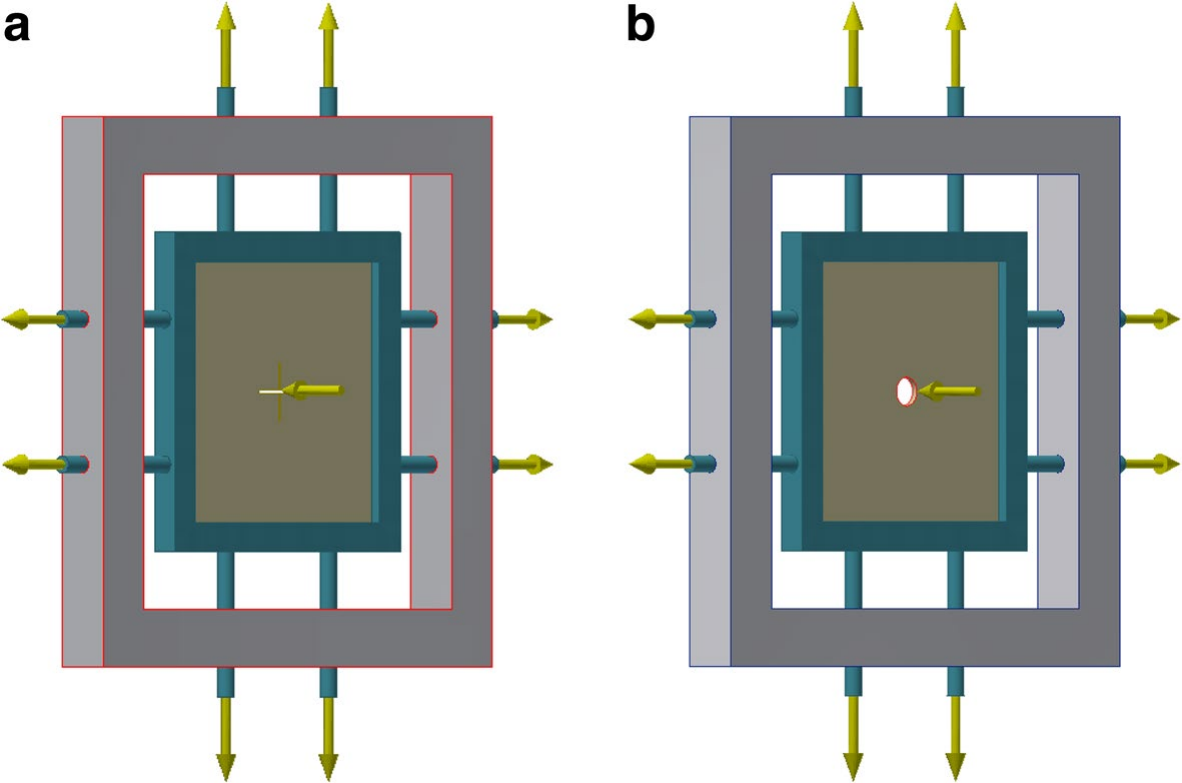
**a**: Simulation design for the traditional setting*:* The traditional setting was generated using a cruciate incision with a length of 20 mm in the simulation-flap. **b**: Simulation setting for the experimental technique. The experimental setting was designed by creating a hole with a 20 mm diameter in the middle of the simulated flap

An in vitro experimental simulation study was performed using AUTODESK Inventor Pro 2016 (Autodesk GmbH, Munich—Germany) [19]. This software enables engineers to turn designs into simulations directly within the Autodest Inventor design environment and display the results in both 2D and 3D [20]. The simulations were performed by a biomedical engineer with expertise in simulations in the period between June and October 2020. A rectangular design was chosen to represent the abdominal wall (fascia). Uniform pressure distribution across the hypothetical fascia was ensured by applying two clamps at each side (Fig. 1a and b). A simulated skin flap of 100 mm × 100 mm with a thickness of 2 mm was assumed in this simulation because of the readily available elasticity data (e.g. modulus of elasticity: 0.3 Mega Pascal) [21].

The experiment was created to stimulate the pressure development over abdominal fascia at the ostomy exit point The circular excision of the fascia at the ostomy site constituted the experimental setting, while the traditionally performed cruciate incision of the fascia was used as control (traditional setting). The traditional setting was generated using a cruciate incision with a length of 20 mm respectively, Fig. 1a. The experimental setting was designed by creating a hole with a 20 mm diameter in the middle of the simulated flap, Fig. 1b. The resulting pressures at the hypothetical ostomy site were recorded both numerical in Mega Pascal (MPa) and visually per color-code; bright colors indicating high pressures. The intra-abdominal pressure was simulated by applying pressure on one side of the setting. Based on the notch effect in material science [22], it is hypothesized that pressure development across the sharp edged of the fascia in the traditional setting would be higher compared to the experimental setting. The outcome of interest was pressure recording over both settings.

## Results

**Fig. 2:**
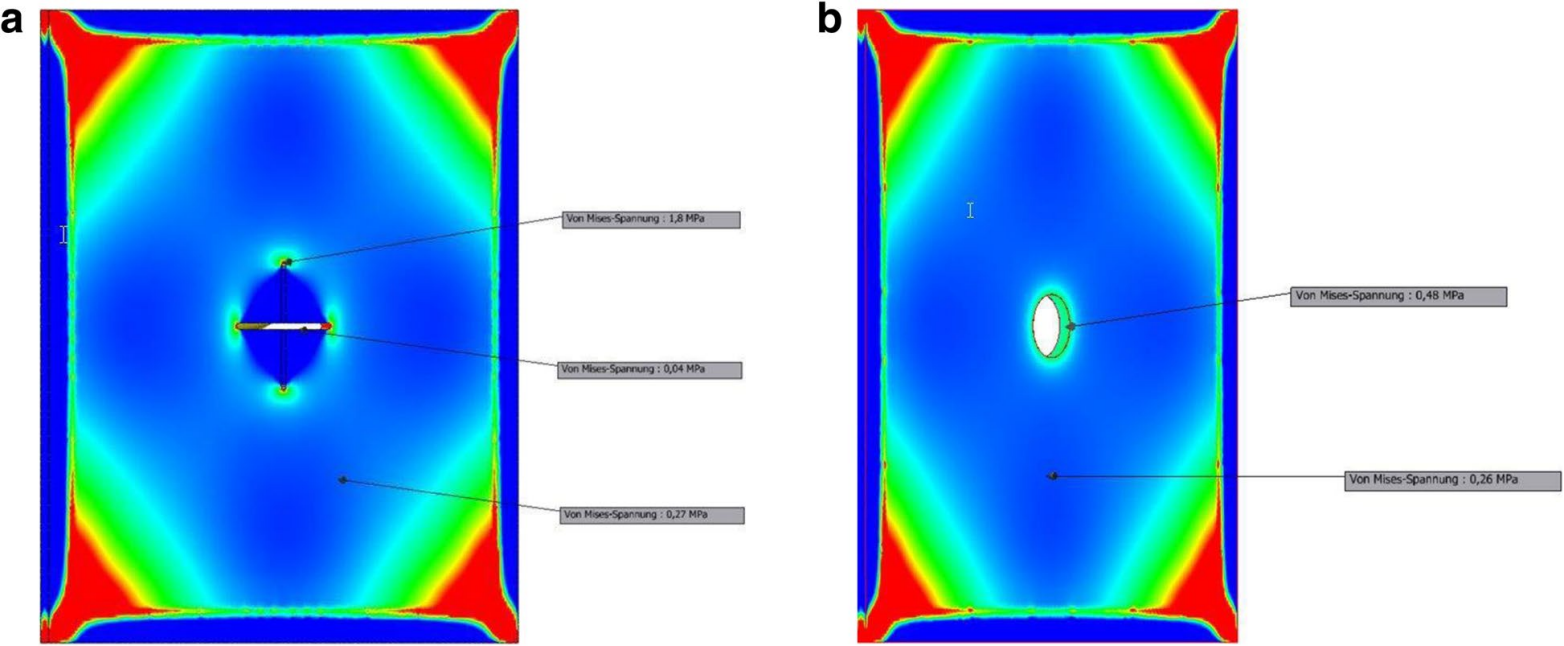
**a**: Baseline pressure distribution across the traditional setting. **b**: Baseline pressure distribution across the experimental setting

**Fig. 3:**
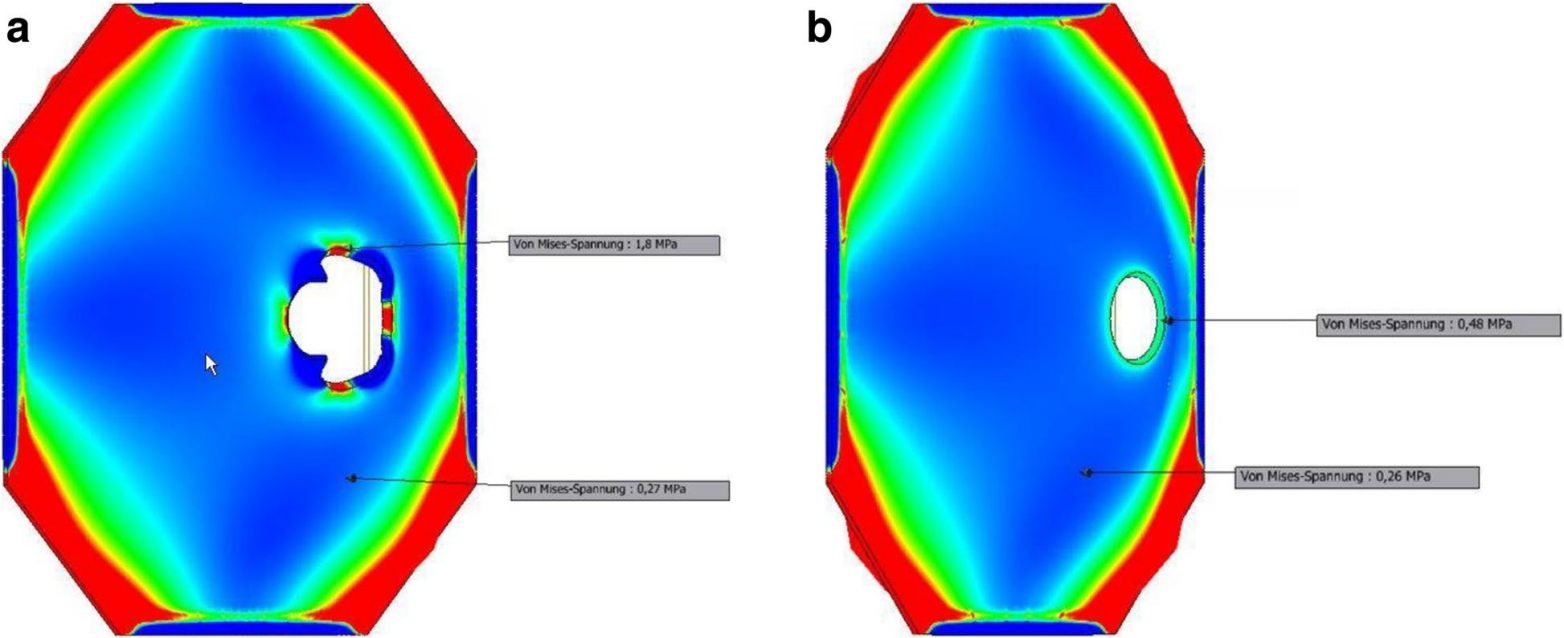
**a**: Pressure distribution across the traditional setting following unilateral pressure application. **b**: Pressure distribution across the experimental setting following unilateral pressure application

The traditional and experimental settings were investigated in this simulation. The pressure values from the simulation are reported using Mega Pascal (MPa) which can be read on the scale on the left. Equally, color-coded pressures recorded in this simulation can be appreciated with brighter colors indication high pressure zones. The high pressures (red color) recorded at the edges of the experimental design in both the traditional and the experimental settings are secondary to the clamps placed at these positions. The pressures recorded over the ostomy site in this simulation was almost four times higher in the traditional setting (1.8 MPa, Fig. 2a) compared to the experimental setting (0.48 MPa, Fig. 2b). This difference was independent of unilateral pressure application e.g., from one surface of the set-up (corresponding to intraabdominal pressure), Fig. 3a and b.

## Discussion

This in vitro experimental simulation study is based on the assumption that high pressure development at the ostomy site following cruciate incision of abdominal fascia for ostomy creation may predispose to the development of PH. Pressure development at ostomy exit site created using two different surgical techniques (cruciate incision vs. circular excision) was compared in this study. The simulations showed an almost four-fold higher pressure at the ostomy site following a cruciate incision (traditional technique) in comparison to a circular excision of the abdominal fascia (experimental setting).

Findings from material physics have identified sharp points including edges of incisions as high pressure zones [23]. This physical knowledge was investigated in this study. The creation of an intestinal ostomy requires exteriorization of a chosen bowel segment via an opening in the abdominal wall. Traditionally, the abdominal fascia is cruciately incised to allow for the free passage of two fingers [18]. The chosen bowel segment is then pulled through this opening to establish the stoma. This technique automatically creates four “sharp edges” in the abdominal wall. This in vitro simulation study confirmed high pressures at the apices of the cruciate incision (red color Fig. 2a). The experimental setting in this study consisted of a circular excision of the abdominal fascia at the ostomy exit point. This technique leaves no “sharp points” or edges. The result is a uniform distribution of pressure across the surface involved. This finding has been termed “notch effect” in material sciences which describes a uniform distribution of pressure over a rounded or curved surface [24].

**Fig. 4:**
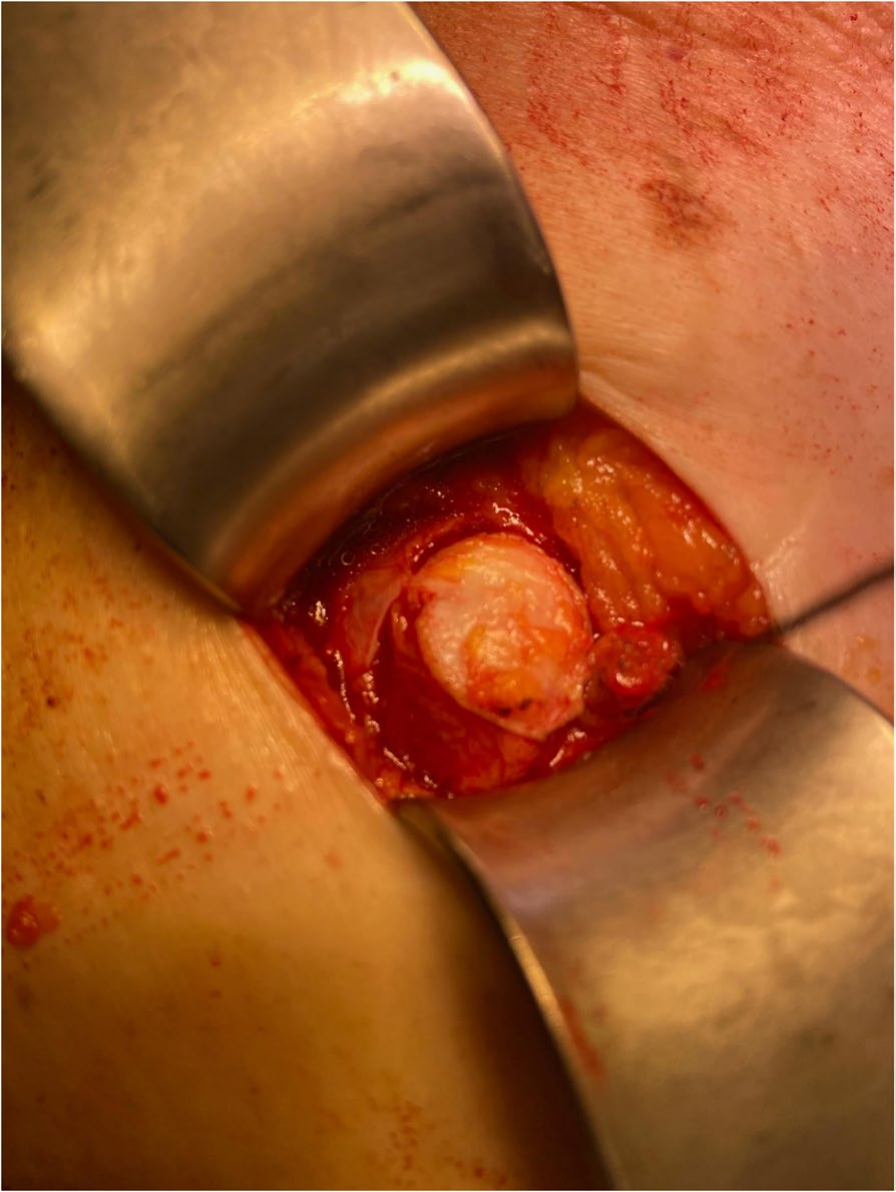
Circular excision of the abdominal fascial to exteriorize the bowel segment for ostomy creation

Using a circular excision to exteriorize the bowel for ostomy creation has previously been described by Semion Resnick [25] and Koltum et al. [17]. A circular cutting device was used for the creation of the ostomy exit site in both reports [17, 25]. Semoin Resnick first tested his circular cutting device on cadavers and later employed the technique in 18 patients. No single PH was observed in his collective after 24 months of follow-up [25]. Using the same technique, Koltum et al. reported a 3% rate of PH in 32 patients after a mean follow-up of 7 years. This is in line with the author´s personal experience with one case of PH in 16 (6.3%) patients with terminal ostomies after a median follow-up of 27 months using the circular excision (Peter C. Ambe, unpublished data). The patient developed a PH 38 months after undergoing proctectomy with the creation of a terminal colostomy due to therapy-refractory Crohn proctitis. An interesting observation from my personal experience is the fact that ostomy takedown is not an issue following the circular excision technique because the small defect created still allows for a tension-free fascia closure. Therefore, this technique (Fig. 4) can be safely employed for the creation of both temporary and permanent stomas, especially because some temporarily created stomas may not be closed [26].

A critical aspect with regard to the cutter-assisted technique used in the papers discussed above [17, 25] in my opinion is the fact that all layers of the abdominal wall including the rectus musculature at the ostomy site were involved. My preference is to leave the rectus muscle intact by performing a blunt dissection and retraction without dividing muscle fibers.

Looking at the pressure data alone, the cruciate incision of abdominal fascia results to almost four times higher pressures compared to the circular excision. Assuming that the pressure magnitude equates to the risk of PH development, this in vitro experimental simulation study would suggest that the risk of PH may be about four times lower with a circular excision of the abdominal fascia at the ostomy exit site compared to the cruciate incision technique. This equates to a 75% risk reduction. An intriguing finding from this in vitro simulation is the fact that pressure development across the experimental ostomy exit site is independent of unilateral pressure application (e.g., Increase in intraabdominal pressure). This finding would mean that PH does not necessarily development as a result of increased abdominal pressure alone. Rather, the surgical technique with regard to the fascia incision/excision seems to be a leading factor in the development of PH.

The limitations to this simulation study are clear. First of all, this is a simulation study and the findings generated in this study need to be further verified. Second, the material-basis of this simulation is a serious limitation. The simulation was based on the vesicoelastic properties of the skin, which are basically different from those of the abdominal wall [27]. Despite the above limitations, the main finding from this simulation study suggests that the surgical technique employed during ostomy creating may significantly influence the development of PH. Generating solid evidence on the efficacy of the excisional technique as a good means of reducing the risk of PH via well-designed studies should be the logical next step.

## Conclusion

The traditional cruciate incision of the abdominal fascia for the creation of an intestinal ostomy predisposes to increased pressures at the incision edges thus increasing the risk of parastomal hernia. This effect is not seen following circular excision of the abdominal fascia. Thus, this surgical aspect may be adopted as a possible means of reducing the risk of parastomal hernia in patients undergoing ostomy surgery.

## Data Availability

The dataset supporting the conclusions of this article is included within the article.

## Abbreviations

Ct: Computed tomography
MPa: Mega Pascal
PH: Parastomal Hernia
QoL: Quality of Life
